# Maternal-fetal adipokine remodeling in obesity-complicated pregnancies: associations with neonatal macrosomia and NICU requirement

**DOI:** 10.3389/fendo.2026.1904956

**Published:** 2026-09-09

**Authors:** Kubra Nur Aslaner, Rauf Melekoglu, Seyma Yasar, Busra Berfin Polat, Ercan Yilmaz, Nese Basak Turkmen

**Affiliations:** 1 Department of Obstetrics and Gynecology, Faculty of Medicine, Inonu University, Malatya, Türkiye; 2 Department of Biostatistics and Medical Informatics, Faculty of Medicine, Inonu University, Malatya, Türkiye; 3 Faculty of Pharmacy, Inonu University, Malatya, Türkiye

**Keywords:** adipokines, adipsin, fetuin-A, fetus, leptin, obesity, pregnancy, retinol-binding protein 4

## Abstract

**Introduction:**

Maternal obesity is associated with altered maternal–fetal adipokine profiles and adverse early neonatal outcomes. This study aimed to characterize maternal and fetal adipokine patterns across obesity severity groups and to evaluate their associations with macrosomia and neonatal intensive care unit (NICU) requirement.

**Methods:**

In this prospective study, 126 singleton pregnancies were stratified into normal-weight, class I obesity, and class II–III obesity groups (n=42 each). Maternal venous and fetal cord blood adiponectin, leptin, asprosin, fetuin-A, retinol-binding protein-4 (RBP-4), adipsin, and glucose transporter-1 (GLUT-1) were assessed at delivery. Macrosomia and NICU requirement were evaluated using group comparisons, receiver operating characteristic (ROC) analyses, and multivariable logistic regression models.

**Results:**

Maternal and fetal adipokine profiles differed significantly across obesity severity groups. Maternal fetuin-A, RBP-4, and adipsin levels, along with fetal leptin, fetuin-A, RBP-4, adipsin, and GLUT-1 levels, increased significantly with increasing obesity severity. In ROC analyses, fetal adipsin showed the highest discrimination for macrosomia (AUC=0.951), whereas fetal RBP-4 showed the highest discrimination for NICU requirement (AUC=0.781). In exploratory adjusted analyses, fetal adipsin remained associated with macrosomia (adjusted OR=5.43, 95% CI 2.14–13.77), whereas fetal RBP-4 remained statistically associated with NICU requirement (adjusted OR=3.31, 95% CI 1.87–5.86).

**Discussion:**

Maternal obesity is accompanied by distinct maternal and fetal adipokine remodeling at delivery, with fetal adipsin and RBP-4 showing outcome-specific associations with neonatal macrosomia and NICU requirement, respectively. The compartment-specific biomarker patterns further suggest that the fetal metabolic response to increasing maternal adiposity is not simply a reflection of maternal circulating profiles. These exploratory findings warrant validation in larger, independent cohorts.

## Graphical Abstract

**Figure:**
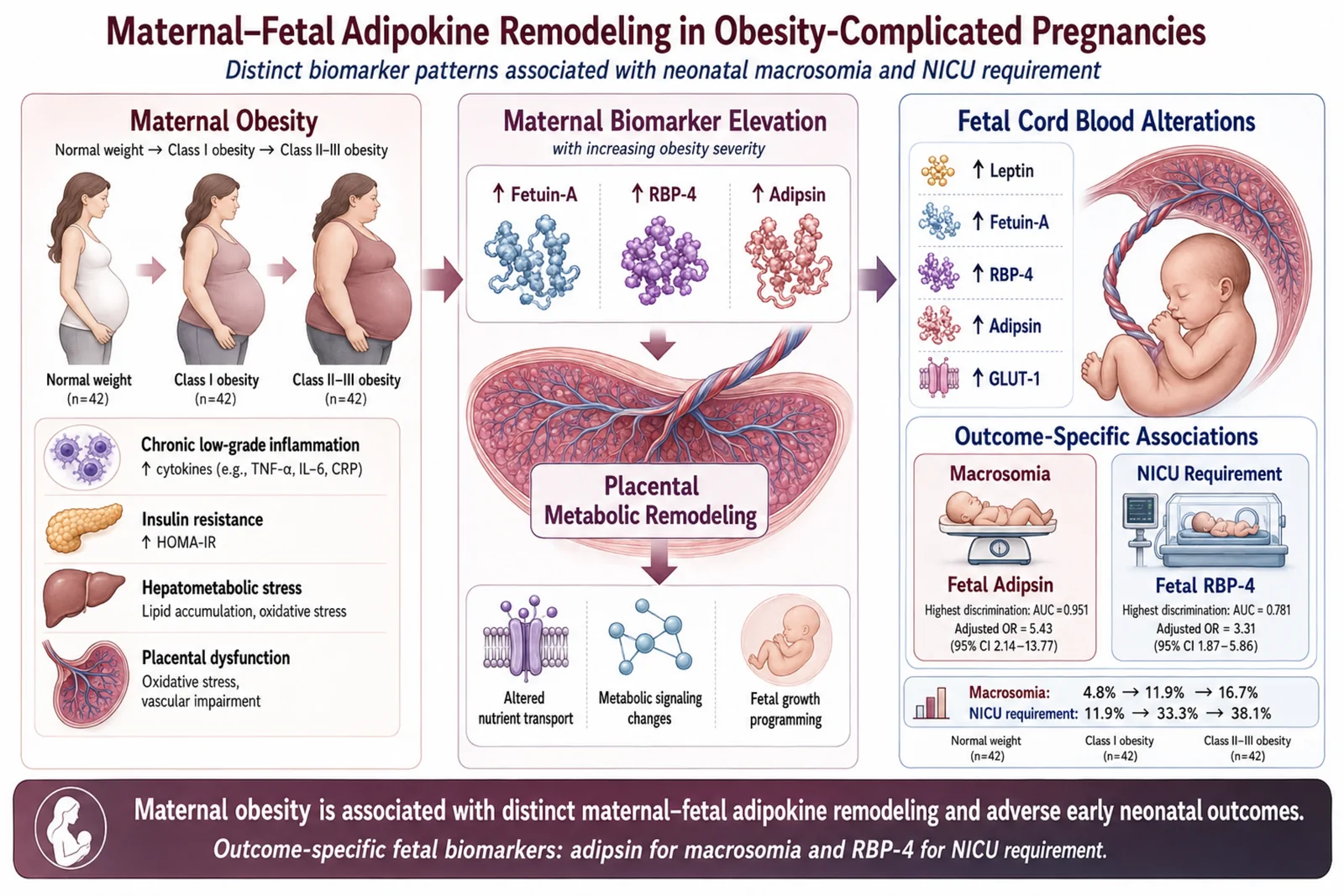

## Introduction

Pregnancy represents one of the most profound physiological and metabolic adaptations in human biology, requiring tightly coordinated alterations in maternal glucose metabolism, lipid homeostasis, inflammatory signaling, endocrine regulation, and energy utilization to support fetal growth and development. Under physiological conditions, these adaptive mechanisms maintain maternal-fetal homeostasis and ensure an optimal intrauterine environment for fetal maturation. However, preexisting metabolic disorders, particularly maternal obesity, may disrupt these finely regulated processes and predispose both the mother and fetus to a broad spectrum of adverse obstetric and neonatal outcomes (1).

Maternal obesity has emerged as one of the most prevalent and clinically significant complications of modern pregnancy, with a steadily increasing global prevalence in both developed and developing countries (2). Obesity-costress andregnancies are characterized by chronic low-grade inflammation, exaggerated insulin resistance, dysregulated lipid metabolism, oxidative stress, and altered adipokine secretion. These metabolic disturbances may affect placental structure and function and have been associated with altered nutrient transport, fetal energy balance, and neonatal outcomes (3). Increasing evidence suggests that the metabolic environment established during fetal life may exert long-term effects on offspring health, forming the basis of the Developmental Origins of Health and Disease hypothesis (4). In this context, maternal obesity has been associated not only with adverse perinatal outcomes but also with an increased lifelong risk of obesity, insulin resistance, type 2 diabetes mellitus, and cardiovascular disease in the offspring (5).

Many of these obesity-associated metabolic disturbances are mediated through adipokines, which constitute a major component of maternal-placental-fetal endocrine communication. Adipose tissue is now recognized as a highly active endocrine organ rather than merely a passive energy storage compartment (6). Beyond its metabolic functions, adipose tissue secretes numerous bioactive molecules, collectively termed adipokines, which play critical roles in inflammatory regulation, insulin sensitivity, glucose metabolism, angiogenesis, placental development, and fetal growth (7). Classical adipokines such as leptin, adiponectin, resistin, and adipsin have been extensively investigated in metabolic diseases and pregnancy-related disorders. During normal pregnancy, maternal adipokine concentrations change dynamically in parallel with gestational adaptations in insulin sensitivity, adipose tissue expansion, placental endocrine activity, and systemic inflammation. Leptin levels generally increase as pregnancy progresses, reflecting both maternal adiposity and placental production, whereas adiponectin tends to decrease, particularly in pregnancies complicated by obesity and insulin resistance. Emerging adipokines and hepatokines, including fetuin-A, RBP-4, adipsin, and asprosin, have also been linked to obesity-related metabolic dysfunction, impaired insulin signaling, lipid metabolism, and inflammatory activation during pregnancy (8, 9). Although postpartum adipokine trajectories remain less consistently defined, available data suggest that the peripartum and early postpartum periods represent metabolically dynamic windows during which obesity-related adipokine alterations may persist or gradually normalize (8). Therefore, evaluation of maternal and fetal adipokine profiles at delivery may provide clinically relevant information regarding the maternal-fetal metabolic milieu at the time of neonatal transition.

Among these emerging biomarkers, fetuin-A, a glycoprotein primarily synthesized by the liver, has been identified as a potent inhibitor of insulin receptor tyrosine kinase activity and has been implicated in the pathogenesis of obesity-related insulin resistance and chronic inflammation (10). Retinol-binding protein-4 (RBP-4) is a metabolically active hepatokine closely linked to glucose intolerance and impaired insulin signaling (11), while adipsin, a component of the alternative complement pathway, has been associated with adipocyte differentiation, lipid accumulation, and metabolic homeostasis (12). Asprosin, a more recently characterized fasting-induced adipokine, has similarly been implicated in hepatic glucose production and insulin resistance (9). Collectively, these hepatokines and adipokines provide complementary insight into the metabolic remodeling that accompanies pregnancies complicated by obesity, extending beyond the classical leptin-adiponectin axis.

Reported circulating adipokine concentrations during pregnancy vary substantially across studies according to gestational age, maternal body mass index (BMI), sample type (maternal versus cord blood), assay platform, antibody specificity, calibration procedure, dilution protocol, and unit reporting (8, 13). Published reference values for leptin, adiponectin, fetuin-A, RBP-4, adipsin, and asprosin therefore differ considerably between cohorts, and few studies have reported paired maternal-fetal concentrations using the same analytical platform (**Supplementary Table 2**). Standardized circulating serum reference values for GLUT-1 remain particularly scarce, as most prior work has assessed placental GLUT-1 expression rather than circulating concentrations (14). This methodological heterogeneity limits direct numerical comparison across studies and underscores the importance of interpreting absolute concentrations within the context of the present cohort rather than as universal reference values.

In obesity-complicated pregnancies, the placenta is continuously exposed to a metabolically adverse intrauterine milieu characterized by hyperglycemia, hyperinsulinemia, dyslipidemia, and systemic inflammation. This altered microenvironment may trigger adaptive fetal responses involving changes in adipogenesis, lipogenesis, glucose transport, and energy utilization, predisposing fetuses of mothers with obesity to excessive fat accumulation, accelerated growth patterns, and neonatal macrosomia. Importantly, the metabolic consequences of maternal obesity extend beyond fetal overgrowth and may also compromise early neonatal adaptation. Neonates born to mothers with obesity exhibit increased risks of hypoglycemia, respiratory distress, thermoregulatory instability, and neonatal intensive care unit (NICU) admission (15), suggesting that intrauterine metabolic stress may substantially influence postnatal physiological transition.

Despite growing interest in adipokine biology during pregnancy, several important gaps remain in the current literature. Most previous studies have primarily focused on maternal circulating biomarkers, whereas relatively few investigations have simultaneously evaluated maternal and fetal adipokine profiles (16). Although recent studies have begun to evaluate paired maternal and cord blood adipokine profiles, most have focused on classical adipokines such as leptin and adiponectin. In contrast, data regarding emerging adipokines and hepatokines, including fetuin-A, RBP-4, adipsin, asprosin, and glucose transport-related biomarkers such as GLUT-1, remain limited in paired maternal-fetal samples. Furthermore, available evidence regarding emerging adipokines such as fetuin-A, RBP-4, adipsin, and asprosin in relation to neonatal outcomes remains limited and inconsistent (17). In particular, the relationship between fetal cord blood adipokine profiles at delivery and early neonatal outcomes has not yet been sufficiently clarified. A comprehensive evaluation of paired maternal and fetal adipokine alterations may therefore provide clinically relevant information regarding the associations between maternal obesity, delivery-time maternal-fetal metabolic profiles, neonatal macrosomia, and NICU requirement.

Accordingly, the present study aimed to investigate maternal and fetal adipokine profiles in pregnancies complicated by obesity and to evaluate their associations with maternal metabolic parameters, neonatal macrosomia, and NICU requirement. By simultaneously analyzing maternal serum and fetal cord blood biomarkers across different obesity severity groups, this study sought to characterize delivery-time maternal-fetal adipokine profile differences and to examine their associations with neonatal macrosomia and NICU requirement. To our knowledge, this is among the first prospective studies to simultaneously evaluate maternal and fetal cord blood profiles of fetuin-A, RBP-4, adipsin, asprosin, leptin, adiponectin, and GLUT-1 across different maternal obesity severity groups in relation to these early neonatal outcomes.

We hypothesized that increasing maternal obesity severity would be associated with distinct maternal and fetal cord blood adipokine alterations at delivery and that corresponding biomarker profiles might differ according to neonatal macrosomia and NICU requirement.

## Materials and methods

### Study design and participants

This prospective observational study was conducted between May 2024 and May 2025 at the Department of Obstetrics and Gynecology, Inonu University Faculty of Medicine, Malatya, Türkiye. Ethical approval was obtained from the Inonu University Scientific Research and Publication Ethics Committee (Approval No: 2024/48, dated April 24, 2024). Written informed consent was obtained from all participants prior to enrollment. The study protocol was conducted in accordance with the principles of the Declaration of Helsinki.

A total of 126 singleton pregnant women who delivered at ≥37 gestational weeks were consecutively recruited and stratified according to pre-pregnancy BMI. Participants were categorized into three groups: normal-weight controls (BMI 18.5-24.9 kg/m², n=42), class I obesity (BMI 30.0-34.9 kg/m², n=42), and class II–III obesity (BMI ≥35.0 kg/m², n=42). To minimize metabolic overlap between study groups and better characterize obesity-related alterations, overweight women (BMI 25.0-29.9 kg/m²) were excluded from the study.

Women aged 18–45 years with reliable pre-pregnancy anthropometric records and singleton live pregnancies were eligible for inclusion. Exclusion criteria included multiple gestation, preeclampsia, gestational diabetes mellitus, pregestational diabetes, chronic hypertension, chronic renal disease, autoimmune disorders, active infection, fetal growth restriction, major fetal anomaly, intrauterine fetal demise, and systemic corticosteroid or immunosuppressive therapy during pregnancy. These criteria were applied to reduce confounding metabolic and inflammatory influences and to more specifically evaluate the independent effects of maternal obesity on adipokine profiles and neonatal outcomes.

### Anthropometric and clinical assessments

Pre-pregnancy weight data were obtained from electronic medical records, primary healthcare databases, or documented first-trimester measurements. Maternal height was measured using a wall-mounted stadiometer with participants standing barefoot in the Frankfurt plane position. Maternal body weight was measured using a calibrated digital scale. BMI was calculated as weight in kilograms divided by height in meters squared (kg/m²). Gestational weight gain was determined by subtracting pre-pregnancy weight from the last recorded weight prior to delivery. Systolic and diastolic blood pressure measurements were obtained before delivery under standardized resting conditions using an automated oscillometric device with an appropriately sized cuff. Measurements were performed after at least 10 minutes of rest in the seated position, and the mean of two consecutive measurements was recorded.

### Maternal blood sampling and processing

Maternal venous blood samples were collected from the antecubital vein before the onset of labor in elective deliveries or during the initial pre-delivery evaluation in urgent admissions. Fasting status was confirmed before sampling, including in urgent admissions, and only samples obtained after a fasting period of at least 8 hours were used for glucose, insulin, and homeostasis model assessment of insulin resistance (HOMA-IR) analyses. Approximately 10 mL of blood was obtained from each participant using serum-separating vacuum tubes. Samples were allowed to clot at room temperature for 20-30 minutes and subsequently centrifuged at 3000 rpm for 10 minutes. Serum aliquots were transferred into labeled polypropylene microtubes and stored at -80 °C until biochemical analyses were performed. To minimize analytical variability, repeated freeze–thaw cycles were avoided.

### Umbilical cord blood collection

Umbilical cord blood samples were obtained immediately after delivery and before placental expulsion. Following double clamping of the umbilical cord, approximately 5-7 mL of blood was aspirated from the umbilical vein using a sterile 21-gauge needle. Cord blood samples were processed under identical laboratory conditions as maternal samples, including clotting, centrifugation, aliquoting, and storage at -80 °C.

### Biochemical and adipokine analyses

Maternal and fetal serum glucose, total cholesterol, triglycerides, high-density lipoprotein cholesterol (HDL-C), and low-density lipoprotein cholesterol (LDL-C) levels were measured using standard automated biochemical analyzers at the central hospital laboratory. LDL-C concentrations were determined either by direct enzymatic measurement or calculated using the Friedewald formula where appropriate.

Insulin resistance was estimated using the HOMA-IR formula:

HOMA-IR = fasting glucose (mg/dL) × fasting insulin (µIU/mL)/405.

Total adiponectin was measured because isoform-specific adiponectin assays were not available within the scope of the study. Maternal serum and fetal cord serum concentrations of total adiponectin, asprosin, leptin, fetuin-A, glucose transporter-1 (GLUT-1), retinol-binding protein-4 (RBP-4), adipsin, and insulin were measured using commercially available enzyme-linked immunosorbent assay (ELISA) kits supplied by A.B.T Laboratory Industry (Ankara, Türkiye), according to the manufacturer’s instructions. Optical density values were measured at 450 nm using a BioTek Synergy HT microplate reader (BioTek Instruments, Winooski, VT, USA). All samples were analyzed in duplicate, and measurements showing excessive intra-assay variability were repeated.

Samples were thawed once immediately before analysis, and repeated freeze–thaw cycles were avoided. To ensure that measured concentrations fell within the assay-specific standard-curve range, maternal and fetal serum samples were diluted at analyte-specific ratios prior to measurement, and the corresponding dilution factor was applied when calculating the final concentration in the original sample. For samples yielding concentrations above the upper limit of the standard curve at the prespecified dilution, the assay was repeated using a higher dilution factor, and the corresponding factor was applied to calculate the final concentration. Assay kit characteristics, standard-curve ranges, and applied dilution factors are summarized in **Supplementary Table 1**, and representative published concentrations are provided in **Supplementary Table 2** for contextual interpretation. Laboratory quality-control procedures were routinely performed throughout the study period.

### Neonatal assessment

Neonatal evaluations were performed immediately after birth by neonatal nursing staff and pediatric residents according to institutional protocols. Birth weight was measured within the first 30 minutes after delivery using a calibrated digital neonatal scale, with measurements recorded to the nearest 10 g. Neonatal length was measured using a standardized infantometer.

Macrosomia was defined as a birth weight ≥4000 g irrespective of gestational age. Neonates were therefore categorized into macrosomic and non-macrosomic groups for comparative analyses. Additional neonatal parameters including umbilical cord pH, base deficit, and NICU admission were recorded. NICU admission was determined according to institutional neonatal care protocols. Indications included respiratory distress requiring monitoring or respiratory support, neonatal hypoglycemia requiring intravenous glucose therapy or close glucose monitoring, suspected sepsis, feeding intolerance, thermoregulatory instability, need for prolonged cardiorespiratory observation, or other neonatal conditions requiring specialist neonatal care. Detailed data regarding the primary indication for NICU admission and length of NICU stay were not systematically collected and were therefore not included in the analyses.

### Sample size calculation

Sample size estimation was based on adiponectin concentrations reported in a previous study evaluating maternal obesity according to BMI categories (16). Assuming an effect size of 0.34, a type I error rate of 5% (α=0.05), and a statistical power of 92.5%, a minimum of 42 participants per group was required according to one-way analysis of variance assumptions, resulting in a total target sample size of 126 participants.

### Statistical analysis

All statistical analyses were performed using IBM SPSS Statistics for Windows, version 26.0 (IBM Corp., Armonk, NY, USA). Normality was assessed using the Shapiro-Wilk test. Three-group comparisons used one-way ANOVA for normally distributed variables and the Kruskal-Wallis test otherwise; two-group comparisons used Welch’s t-test or the Mann–Whitney U test, as appropriate. Categorical variables were analyzed using the chi-square or Fisher’s exact test. ROC analyses reported AUCs with 95% confidence intervals and Youden-index cutoffs. All outcome-specific two-group comparisons, ROC analyses, and multivariable logistic regression models were performed in the full cohort of 126 participants. Because of the limited number of outcome events, the number of covariates in the multivariable models was limited *a priori*. Pre-pregnancy BMI was selected as the primary adjustment covariate because it defined the obesity-severity groups and was therefore considered the most clinically relevant potential confounder of biomarker–outcome associations. The NICU model included pre-pregnancy BMI, maternal fetuin-A, fetal RBP-4, and fetal GLUT-1, whereas the macrosomia model included pre-pregnancy BMI, maternal fetuin-A, fetal adipsin, and fetal GLUT-1. Odds ratios with 95% confidence intervals were reported. Analyses were exploratory, and a two-sided p<0.05 was considered statistically significant. Exploratory associations among pre-pregnancy BMI, selected maternal and fetal biomarkers, birth weight, macrosomia, and NICU requirement were summarized using Spearman rank-correlation coefficients and displayed as a correlation matrix.

**Figure 1:**
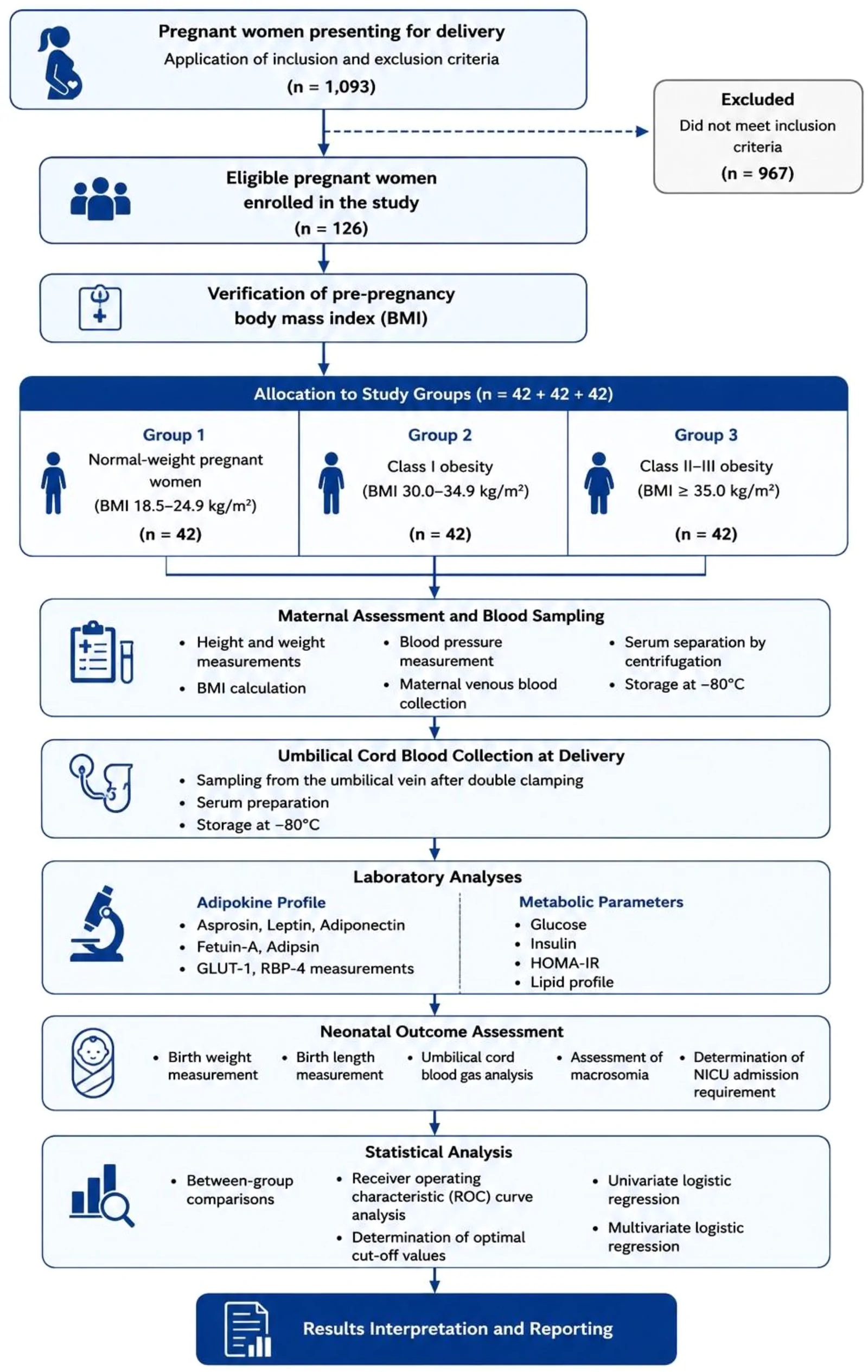
Study design and analytical workflow. Overview of participant recruitment, study group allocation, maternal and fetal biospecimen collection, biomarker analyses, neonatal outcome assessment, and statistical modeling procedures.

A schematic overview of the study design and analytical workflow is presented in **Figure 1**.

## Results

### Study population and baseline characteristics

A total of 126 singleton pregnancies were included in the final analysis and stratified according to pre-pregnancy body mass index (BMI) into three groups: normal-weight controls (BMI 18.5-24.9 kg/m², n=42), class I obesity (BMI 30.0-34.9 kg/m², n=42), and class II-III obesity (BMI ≥35 kg/m², n=42). A total of 252 paired maternal venous and fetal cord blood serum samples were analyzed.

Baseline demographic and obstetric characteristics are summarized in **Table 1**. Gestational age at sampling and delivery did not differ significantly among the study groups (p=0.952), confirming comparable obstetric timing across cohorts. Maternal age, gravida, and parity were also similar between groups (all p>0.05). As expected, pre-pregnancy BMI and gestational weight gain increased progressively across obesity categories (both p<0.001). Diastolic blood pressure differed significantly among groups (p=0.034), whereas systolic blood pressure, mode of delivery, and cesarean indications were not significantly different.

**Table 1:** Maternal demographic and obstetric characteristics according to pre-pregnancy body mass index category.

Characteristic	Normal Weight(n = 42)	Class I Obesity(n = 42)	Class II-III Obesity(n = 42)	*p* value
Maternal age (years)	29.17 ± 4.67	31.62 ± 5.86	30.50 ± 4.86	0.307
Gestational age at sampling (weeks)	38.64 ± 1.01	38.60 ± 1.08	38.67 ± 1.22	0.952
Gravidity	2.17 ± 1.31	2.45 ± 1.56	2.74 ± 1.45	0.169
Parity	0.83 ± 1.06	1.19 ± 1.38	1.24 ± 1.19	0.245
Pre-pregnancy BMI (kg/m²)	21.29 ± 1.41	30.98 ± 0.86	37.10 ± 1.89	***<0.001***
Gestational weight gain (kg)	9.48 ± 2.31	10.62 ± 1.89	13.40 ± 3.32	***<0.001***
Systolic blood pressure (mmHg)	110.0 ± 8.6	116.26 ± 8.47	115.79 ± 11.53	0.071
Diastolic blood pressure (mmHg)	72.57 ± 6.57	75.05 ± 9.00	77.02 ± 9.07	***0.034***
Mode of delivery, n (%) - p = 0.860				
Vaginal delivery	3 (7.1)	2 (4.8)	2 (4.8)	
Cesarean delivery	39 (92.9)	40 (95.2)	40 (95.2)	
Primary indication for cesarean delivery, n (%) - p = 0.169				
Previous cesarean section	17 (43.6)	16 (40.0)	17 (42.5)	
Cephalopelvic disproportion	11 (28.2)	11 (27.5)	6 (15.0)	
Fetal distress	4 (10.3)	3 (7.5)	6 (15.0)	
Malpresentation	5 (12.8)	2 (5.0)	5 (12.5)	
Arrest of labor progression	2 (5.1)	5 (12.5)	0 (0.0)	
Suspected LGA fetus	0 (0.0)	3 (7.5)	5 (12.5)	
Placental abnormality	0 (0.0)	0 (0.0)	1 (2.5)	

Data are presented as mean ± standard deviation (SD) or number (percentage), as appropriate. Group comparisons were performed using one-way ANOVA for continuous variables and χ² test for categorical variables. A two-sided *p* < 0.05 was considered statistically significant. Significant *p* values are indicated in bold italics. BMI, body mass index; LGA, large for gestational age.

### Neonatal outcomes according to maternal obesity severity

Neonatal and delivery-related outcomes are presented in **Table 2**. Birth weight increased significantly with increasing maternal obesity severity (p<0.001). Neonates born to women with class II-III obesity had significantly higher birth weights compared with both normal-weight controls and class I obesity groups. In contrast, neonatal length, cord blood pH, and base deficit values were comparable among groups.

**Table 2:** Obstetric and neonatal outcomes according to pre-pregnancy BMI category.

Variable	Normal weight (BMI 18.5-24.9 kg/m²) (n = 42)	Class I obesity (BMI 30.0-34.9 kg/m²) (n = 42)	Class II-III obesity (BMI ≥35 kg/m²) (n = 42)	*p* value
Gestational age at delivery (weeks)	38.64 ± 1.01	38.60 ± 1.08	38.67 ± 1.22	0.952
Birth weight (g)	3182.98 ± 422.46	3359.24 ± 453.90	3758.19 ± 613.35	***<0.001***
Birth length (cm)	49.76 ± 2.01	50.17 ± 1.71	50.81 ± 2.17	0.066
Umbilical cord pH	7.31 ± 0.04	7.29 ± 0.05	7.30 ± 0.04	0.108
Base deficit (mmol/L)	4.17 ± 1.36	4.54 ± 1.47	4.79 ± 1.54	0.161
Macrosomia, n (%)	2 (4.8)	5 (11.9)	7 (16.7)	***0.031***
NICU admission, n (%)	5 (11.9)	14 (33.3)	16 (38.1)	***0.017***

Data are presented as mean ± standard deviation (SD) or n (%), as appropriate. Continuous variables were compared using one-way analysis of variance (ANOVA), whereas categorical variables were compared using the χ² test or Fisher’s exact test, as appropriate. Macrosomia was defined as birth weight ≥4000 g irrespective of gestational age. NICU admission indicates neonatal intensive care unit requirement during the early postnatal period. A two-sided *p* < 0.05 was considered statistically significant. Significant *p* values are indicated in bold italics. BMI, body mass index; NICU, neonatal intensive care unit.

**Figure 2:**
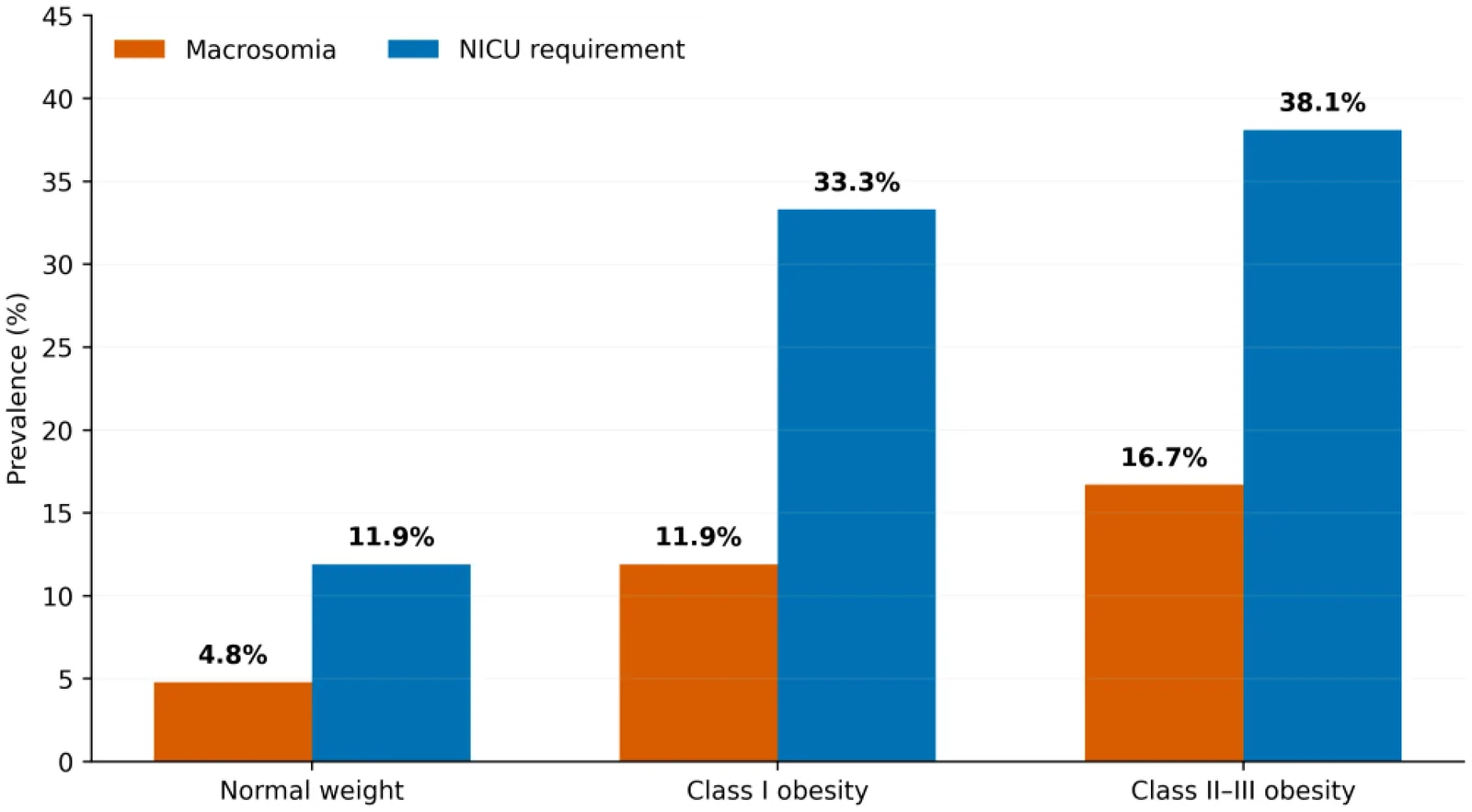
Neonatal outcomes according to maternal obesity severity. Prevalence of neonatal macrosomia and NICU requirement across normal-weight, class I obesity, and class II-III obesity groups, corresponding to **Table 2**.

Macrosomia prevalence increased from 4.8% (2/42) in normal-weight pregnancies to 11.9% (5/42) in class I obesity and 16.7% (7/42) in class II-III obesity (p=0.031). NICU requirement similarly increased from 11.9% to 33.3% and 38.1%, respectively (p=0.017). These outcome frequencies are illustrated in **Figure 2**.

### Maternal metabolic parameters and adipokine profiles

Maternal metabolic and adipokine parameters are summarized in **Table 3**. Maternal fetuin-A, RBP-4, and adipsin concentrations demonstrated a significant stepwise increase across obesity categories (all p<0.001). The highest concentrations of these biomarkers were observed in the class II-III obesity group.

**Table 3:** Maternal metabolic and adipokine profiles according to pre-pregnancy BMI category.

Variable	Normal weight (BMI 18.5-24.9 kg/m²) (n = 42)	Class I obesity (BMI 30.0-34.9 kg/m²) (n = 42)	Class II-III obesity (BMI ≥35 kg/m²) (n = 42)	*p* value
Metabolic parameters				
Glucose (mg/dL)	75.24 ± 12.27	76.19 ± 12.20	79.60 ± 13.61	0.221
Insulin (µIU/mL)	13.77 ± 15.12	16.31 ± 18.08	19.14 ± 19.14	0.179
HOMA-IR	2.78 ± 3.64	3.43 ± 4.18	4.06 ± 4.47	0.085
Total cholesterol (mg/dL)	227.93 ± 60.17	213.12 ± 56.28	208.98 ± 68.27	0.166
LDL cholesterol (mg/dL)	119.77 ± 41.04	105.67 ± 38.33	108.19 ± 42.68	0.148
HDL cholesterol (mg/dL)	64.86 ± 22.36	60.60 ± 15.30	58.86 ± 18.17	0.633
LDL/HDL ratio	1.95 ± 0.84	1.79 ± 0.64	1.92 ± 0.85	0.737
Triglycerides (mg/dL)	217.76 ± 81.76	228.50 ± 100.21	229.12 ± 112.55	0.953
Adipokines and metabolic biomarkers				
Adiponectin (ng/mL)	7,300 ± 125	7,250 ± 125	7,213 ± 125	0.006
Leptin (pg/mL)	42,000 ± 28,000	44,500 ± 30,000	40,950 ± 27,000	0.841
Asprosin (ng/mL)	11.01 ± 6.56	10.27 ± 10.37	9.92 ± 7.14	0.354
Fetuin-A (ng/mL)	474,077 ± 46,140	533,029 ± 28,637	649,680 ± 59,992	***<0.001***
RBP-4 (ng/mL)	25.88 ± 1.47	34.40 ± 2.50	41.74 ± 3.05	***<0.001***
Adipsin (ng/mL)	321.13 ± 67.98	358.81 ± 26.46	420.26 ± 56.58	***<0.001***
GLUT-1 (ng/mL)	2.82 ± 1.13	2.59 ± 0.96	2.35 ± 1.01	0.107

Data are presented as mean ± standard deviation (SD). Three-group comparisons were performed using one-way analysis of variance (ANOVA) or the Kruskal-Wallis test, as appropriate; *post hoc* comparisons were applied where indicated. A two-sided *p* < 0.05 was considered statistically significant. Significant *p* values are indicated in bold italics. HOMA-IR, homeostatic model assessment of insulin resistance; LDL, low-density lipoprotein; HDL, high-density lipoprotein; RBP-4, retinol-binding protein-4; GLUT-1, glucose transporter-1.

Maternal adiponectin levels showed a modest but significant reduction in obese pregnancies compared with controls (p=0.006). In contrast, maternal leptin, asprosin, and GLUT-1 concentrations did not differ significantly among groups. Similarly, maternal glucose, insulin, HOMA-IR, total cholesterol, LDL-C, HDL-C, LDL/HDL ratio, and triglyceride levels showed no statistically significant intergroup differences. In contrast to conventional metabolic parameters, which remained largely comparable across obesity severity categories, several maternal adipokines including fetuin-A, RBP-4, adipsin, and adiponectin demonstrated significant alterations.

### Fetal cord blood adipokine and metabolic profiles

Fetal cord blood biomarker levels differed significantly across maternal obesity severity groups (**Table 4**). Concentrations of fetal leptin, fetuin-A, RBP-4, adipsin, and GLUT-1 increased significantly with increasing maternal obesity severity (all p < 0.001), whereas fetal adiponectin and asprosin did not differ significantly across groups.

**Table 4:** Fetal cord blood metabolic and adipokine profiles according to pre-pregnancy BMI category.

Variable	Normal weight (BMI 18.5–24.9 kg/m²) (n = 42)	Class I obesity (BMI 30.0–34.9 kg/m²) (n = 42)	Class II-III obesity (BMI ≥35 kg/m²) (n = 42)	*p* value
Metabolic parameters				
Glucose (mg/dL)	58.38 ± 10.30	60.55 ± 14.03	64.52 ± 19.47	0.107
Insulin (µIU/mL)	4.63 ± 4.09	4.68 ± 3.72	4.84 ± 4.72	0.917
HOMA-IR	0.81 ± 0.81	0.86 ± 1.18	0.84 ± 0.96	0.981
Total cholesterol (mg/dL)	77.64 ± 43.03	67.29 ± 21.35	78.64 ± 51.00	0.661
LDL cholesterol (mg/dL)	36.51 ± 24.62	30.59 ± 14.22	41.23 ± 30.66	0.527
HDL cholesterol (mg/dL)	30.07 ± 13.51	27.49 ± 10.78	25.64 ± 13.53	0.278
LDL/HDL ratio	1.63 ± 2.43	1.48 ± 1.65	1.82 ± 1.41	0.150
Triglycerides (mg/dL)	39.14 ± 57.19	35.05 ± 27.48	48.02 ± 76.19	0.262
Adipokines and metabolic biomarkers				
Adiponectin (ng/mL)	28,000 ± 850	27,950 ± 900	27,797 ± 1,300	0.357
Leptin (pg/mL)	10,373 ± 4,728	17,652 ± 1,341	22,745 ± 2,787	***<0.001***
Asprosin (ng/mL)	9.53 ± 8.06	10.03 ± 5.19	9.20 ± 5.76	0.382
Fetuin-A (ng/mL)	663,515 ± 33,048	824,557 ± 81,439	872,594 ± 35,977	***<0.001***
RBP-4 (ng/mL)	11.00 ± 1.35	12.50 ± 1.35	14.13 ± 1.35	***<0.001***
Adipsin (ng/mL)	163.03 ± 42.16	182.09 ± 14.51	202.67 ± 13.36	***<0.001***
GLUT-1 (ng/mL)	4.39 ± 2.04	7.33 ± 1.42	9.85 ± 2.17	***<0.001***

Data are presented as mean ± standard deviation (SD). Three-group comparisons were performed using one-way ANOVA or the Kruskal-Wallis test, as appropriate; *post hoc* comparisons were applied where indicated. A two-sided *p* < 0.05 was considered statistically significant. Significant *p* values are indicated in bold italics. HOMA-IR, homeostatic model assessment of insulin resistance; LDL, low-density lipoprotein; HDL, high-density lipoprotein; RBP-4, retinol-binding protein-4; GLUT-1, glucose transporter-1.

### Exploratory correlation analysis

**Figure 3:**
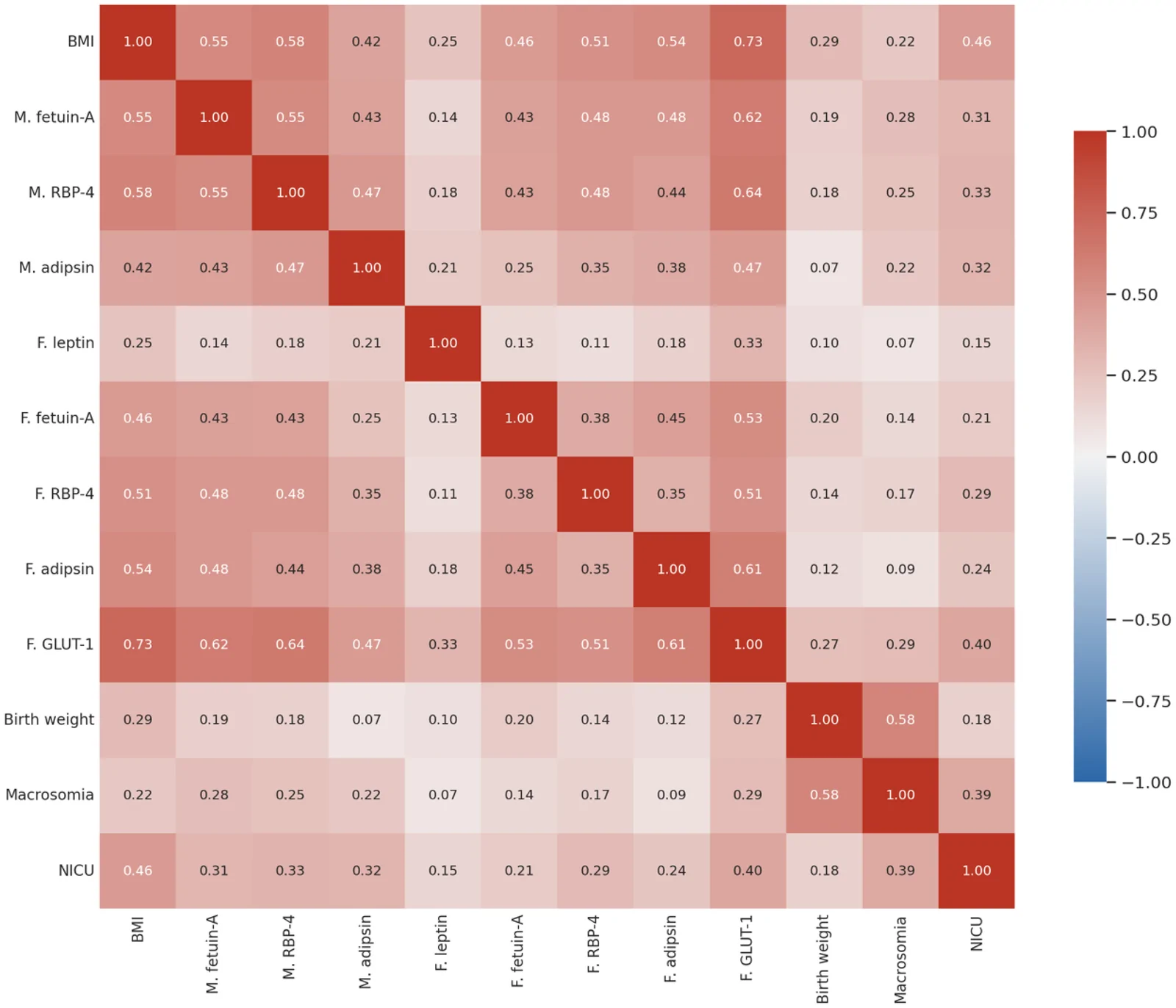
Exploratory Spearman correlation matrix of pre-pregnancy BMI, selected maternal and fetal biomarkers, birth weight, neonatal macrosomia, and NICU requirement. Cell values represent Spearman correlation coefficients (ρ). M., maternal; F., fetal cord blood; BMI, body mass index; NICU, neonatal intensive care unit.

The exploratory Spearman correlation matrix demonstrated that the strongest overall association was between pre-pregnancy BMI and fetal GLUT-1 (ρ=0.73). Among biomarker pairs, maternal RBP-4 was most strongly correlated with fetal GLUT-1 (ρ=0.64), followed by maternal fetuin-A with fetal GLUT-1 (ρ=0.62) and fetal adipsin with fetal GLUT-1 (ρ=0.61). Birth weight was correlated with macrosomia (ρ=0.58), while macrosomia and NICU requirement were moderately correlated (ρ=0.39) (**Figure 3**).

Fetal conventional metabolic parameters, including glucose, insulin, HOMA-IR, total cholesterol, LDL cholesterol, HDL cholesterol, LDL/HDL ratio, and triglyceride concentrations, did not differ significantly across maternal obesity categories.

### Maternal and fetal cord blood biomarkers associated with NICU requirement

In the full cohort, 35 neonates required NICU care (**Table 5**). Maternal glucose concentrations were higher in the NICU group than in the no-NICU group (82.37 ± 11.39 vs 75.41 ± 13.21 mg/dL; p=0.027), whereas maternal HDL cholesterol concentrations were lower (54.13 ± 19.00 vs 62.83 ± 14.58 mg/dL; p=0.034). Maternal fetuin-A (596,570 ± 77,630 vs 535,220 ± 56,160 ng/mL; p<0.001), RBP-4 (35.81 ± 3.40 vs 33.31 ± 4.28 ng/mL; p=0.019), and adipsin (384.23 ± 49.13 vs 360.00 ± 50.09 ng/mL; p=0.009) concentrations were also higher in the NICU group.

**Table 5:** Maternal and fetal cord blood metabolic and adipokine profiles according to NICU requirement in the full study cohort.

Variable	No NICU Requirement (n = 91)	NICU Requirement (n = 35)	*p* value
Maternal biomarkers			
Glucose (mg/dL)	75.41 ± 13.21	82.37 ± 11.39	0.027
Insulin (µIU/mL)	15.78 ± 17.52	18.04 ± 17.01	0.563
HOMA-IR	3.25 ± 3.95	3.88 ± 3.92	0.471
HDL cholesterol (mg/dL)	62.83 ± 14.58	54.13 ± 19.00	0.034
Adiponectin (ng/mL)	7,260 ± 130	7,240 ± 140	0.469
Leptin (pg/mL)	42,140 ± 27,460	43,380 ± 30,710	0.989
Fetuin-A (ng/mL)	535,220 ± 56,160	596,570 ± 77,630	***<0.001***
RBP-4 (ng/mL)	33.31 ± 4.28	35.81 ± 3.40	***0.019***
Adipsin (ng/mL)	360.00 ± 50.09	384.23 ± 49.13	***0.009***
GLUT-1 (ng/mL)	2.54 ± 1.93	2.70 ± 1.95	0.805
Fetal cord blood biomarkers			
Glucose (mg/dL)	59.42 ± 15.19	65.65 ± 20.95	0.118
Insulin (µIU/mL)	4.58 ± 3.88	5.18 ± 4.83	0.532
HOMA-IR	0.82 ± 0.99	0.96 ± 1.12	0.569
HDL cholesterol (mg/dL)	28.44 ± 12.16	25.90 ± 13.82	0.382
Leptin (pg/mL)	16,040 ± 2,000	19,220 ± 3,060	***<0.001***
Asprosin (ng/mL)	10.11 ± 5.58	8.72 ± 5.22	0.199
Fetuin-A (ng/mL)	772,980 ± 64,420	823,050 ± 43,980	***<0.001***
RBP-4 (ng/mL)	11.91 ± 1.33	14.18 ± 2.02	***<0.001***
Adipsin (ng/mL)	179.16 ± 13.22	191.53 ± 18.95	***0.003***
GLUT-1 (ng/mL)	6.48 ± 2.86	9.04 ± 2.45	***<0.001***

Data are presented as mean ± standard deviation (SD). Comparisons were performed using the independent-samples t-test or Mann-Whitney U test, as appropriate. A two-sided *p* < 0.05 was considered statistically significant. Significant *p* values are indicated in bold italics. HDL, high-density lipoprotein; HOMA-IR, homeostatic model assessment of insulin resistance; NICU, neonatal intensive care unit; RBP-4, retinol-binding protein-4; GLUT-1, glucose transporter-1.

Fetal concentrations of leptin (19,220 ± 3,060 vs. 16,040 ± 2,000 pg/mL), fetuin-A (823,050 ± 43,980 vs. 772,980 ± 64,420 ng/mL), RBP-4 (14.18 ± 2.02 vs. 11.91 ± 1.33 ng/mL), adipsin (191.53 ± 18.95 vs. 179.16 ± 13.22 ng/mL), and GLUT-1 (9.04 ± 2.45 vs. 6.48 ± 2.86 ng/mL) were significantly higher among neonates requiring NICU care than among those not requiring NICU care (all p ≤ 0.003).

### Diagnostic performance of maternal and fetal cord blood biomarkers for NICU requirement

**Figure 4:**
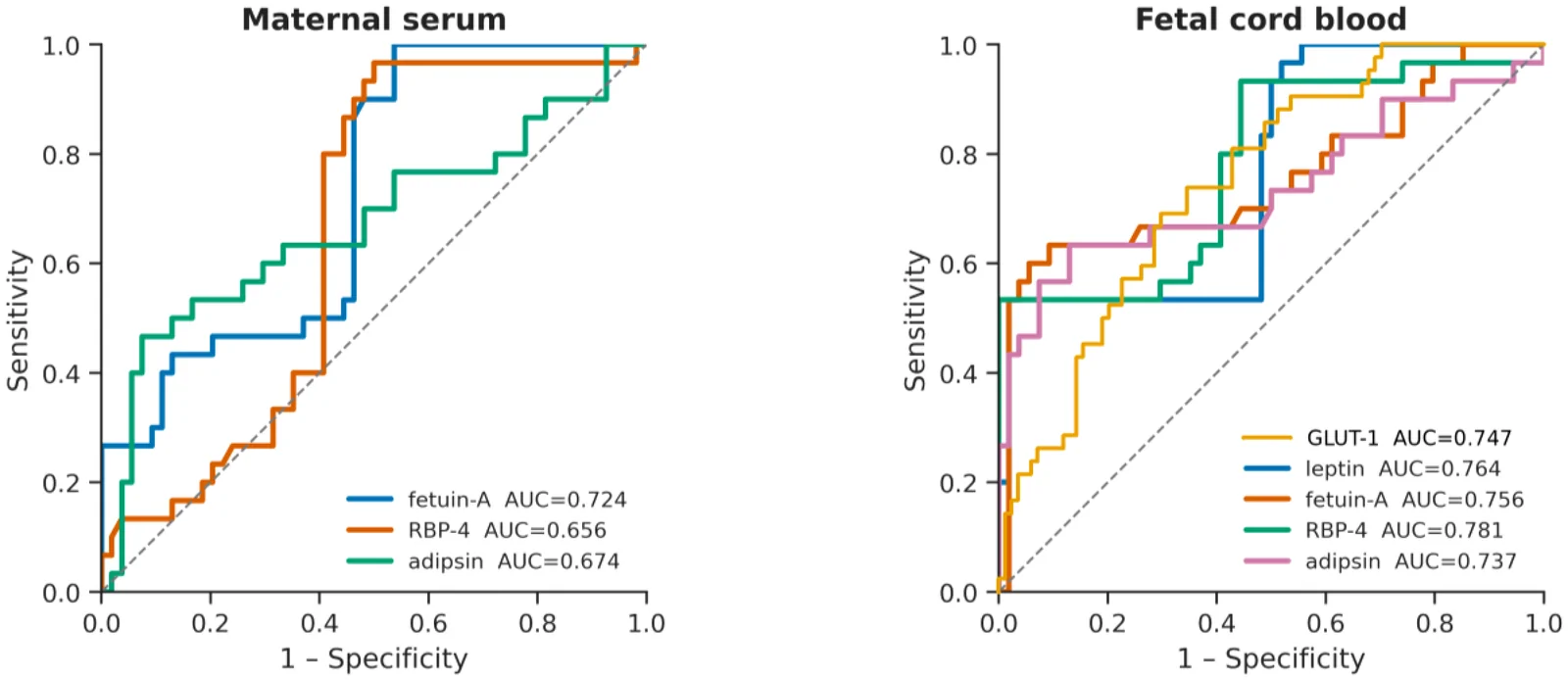
Receiver operating characteristic curves for selected maternal and fetal biomarkers and NICU requirement. Fetal RBP-4 showed the highest AUC among the evaluated fetal biomarkers.

ROC results for NICU requirement are summarized in **Table 6** and **Figure 4**. Among maternal biomarkers, fetuin-A showed the highest discriminatory performance for NICU requirement (AUC = 0.724), followed by adipsin (AUC = 0.674) and RBP-4 (AUC = 0.656). Among fetal biomarkers, RBP-4 yielded the highest AUC (0.781), followed by leptin (0.764), fetuin-A (0.756), GLUT-1 (0.747), and adipsin (0.737), with all fetal biomarkers demonstrating statistically significant discriminatory ability.

**Table 6:** Discriminatory performance of maternal and fetal cord blood biomarkers for NICU requirement.

Biomarker	Source	Cut-off	Sensitivity (%)	Specificity (%)	AUC (95% CI)	*p* value
Fetuin-A	Maternal serum	>508,840	100.0	46.3	0.724 (0.601-0.824)	***<0.001***
RBP-4	Maternal serum	>32.69	96.7	50.0	0.656 (0.539-0.763)	***0.015***
Adipsin	Maternal serum	>396.30	46.7	92.6	0.674 (0.528-0.798)	***0.006***
Leptin	Fetal cord blood	>19,950	53.3	98.1	0.764 (0.647-0.862)	***<0.001***
Fetuin-A	Fetal cord blood	>824,760	60.0	94.4	0.756 (0.620-0.871)	***<0.001***
RBP-4	Fetal cord blood	>13.97	53.3	100.0	0.781 (0.669-0.875)	***<0.001***
Adipsin	Fetal cord blood	>195.27	63.3	87.0	0.737 (0.602-0.858)	***<0.001***
GLUT-1	Fetal cord blood	>7.46	73.8	65.5	0.747 (0.658-0.830)	***<0.001***

AUC, area under the curve; CI, confidence interval; GLUT-1, glucose transporter-1; NICU, neonatal intensive care unit; RBP-4, retinol-binding protein-4. A two-sided *p* < 0.05 was considered statistically significant. Significant *p* values are indicated in bold italics.

### Multivariable logistic regression model for NICU requirement

**Figure 5:**
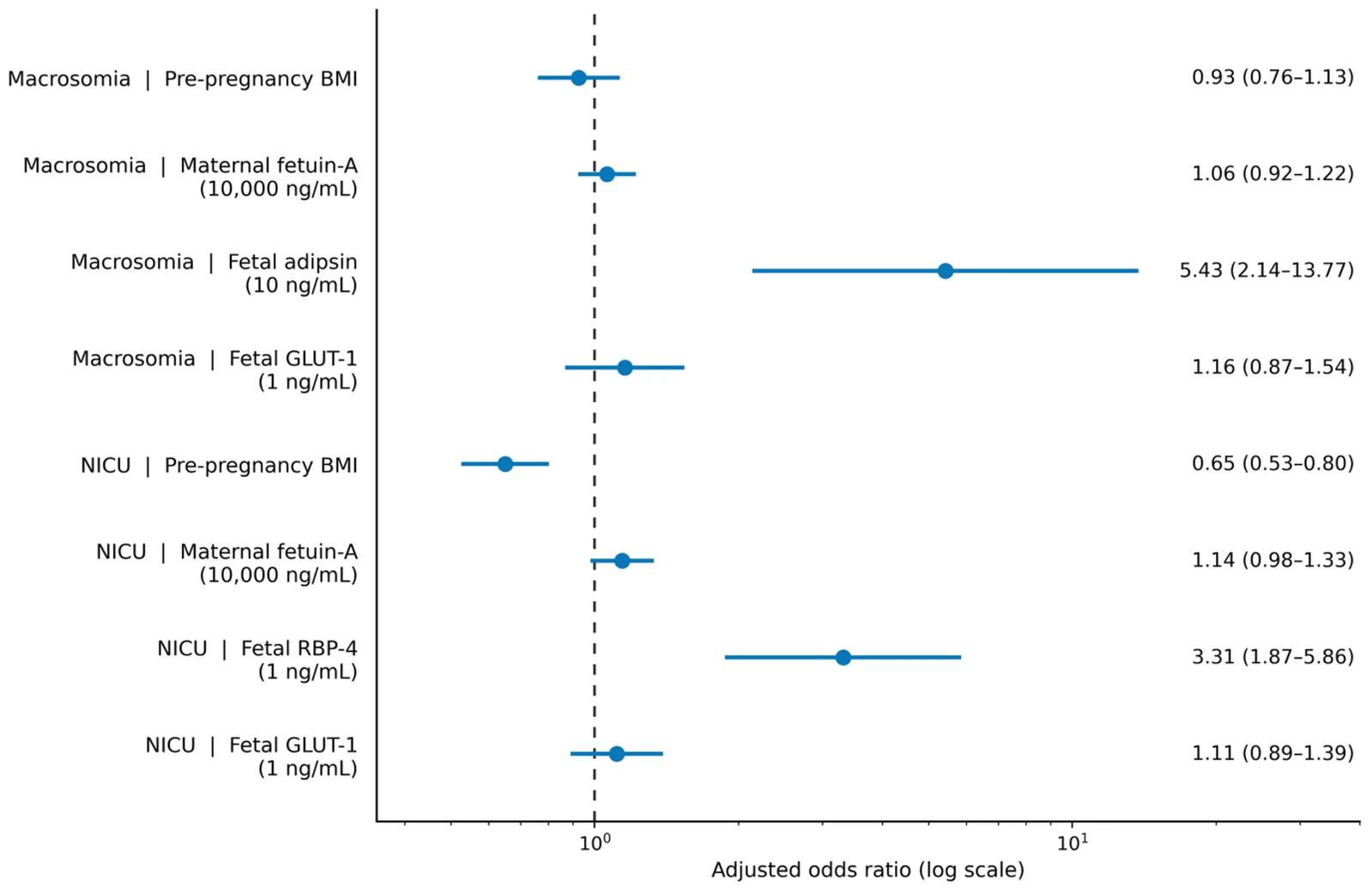
Multivariable logistic regression models for neonatal macrosomia and NICU requirement. Adjusted odds ratios (95% CIs, logarithmic scale) are shown for pre-pregnancy BMI, maternal fetuin-A, fetal adipsin, and fetal GLUT-1 in the macrosomia model and for pre-pregnancy BMI, maternal fetuin-A, fetal RBP-4, and fetal GLUT-1 in the NICU model.

As shown in **Table 7** and **Figure 5**, fetal RBP-4 remained statistically associated with NICU requirement after adjustment (adjusted OR = 3.313 per 1-ng/mL increase, 95% CI 1.874-5.858; p<0.001). In contrast, maternal fetuin-A and fetal GLUT-1 were no longer statistically significant after simultaneous adjustment (adjusted OR = 1.142 per 10,000-ng/mL increase, 95% CI 0.980-1.330; p=0.089 and adjusted OR = 1.113 per 1-ng/mL increase, 95% CI 0.891-1.390; p=0.347, respectively). The inverse adjusted association between pre-pregnancy BMI and NICU requirement should be interpreted cautiously in view of the correlations among BMI and the biomarkers included in the model.

**Table 7:** Univariate and multivariable logistic regression analyses of maternal and fetal biomarkers in relation to NICU admission in the full study cohort.

Predictor	Unadjusted OR (95% CI)	*p* value	Adjusted OR (95% CI)	*p* value
Pre-pregnancy BMI	–	*-*	0.650 (0.526-0.803)	***<0.001***
Maternal fetuin-A (per 10,000 ng/mL)	1.153 (1.063-1.250)	***<0.001***	1.142 (0.980-1.330)	0.089
Fetal RBP-4 (per 1 ng/mL)	2.24 (1.55-3.24)	***<0.001***	3.313 (1.874-5.858)	***<0.001***
Fetal GLUT-1 (per 1 ng/mL)	1.400 (1.198-1.637)	***<0.001***	1.113 (0.891-1.390)	0.347

Values are odds ratios (95% confidence intervals), followed by *p* values. The multivariable NICU model included pre-pregnancy BMI, maternal fetuin-A, fetal RBP-4, and fetal GLUT-1. Biomarker odds ratios are expressed as per increment stated in the table. A two-sided *p* < 0.05 was considered statistically significant. BMI, body mass index; CI, confidence interval; NICU, neonatal intensive care unit; OR, odds ratio; RBP-4, retinol-binding protein-4; GLUT-1, glucose transporter-1. Bold values indicate statistical significance at p < 0.05.

### Maternal and fetal cord blood biomarkers associated with neonatal macrosomia

Maternal and fetal biomarker profiles were compared according to neonatal macrosomia in the full cohort (14 macrosomic and 112 non-macrosomic neonates; **Table 8**). Maternal fetuin-A (631,101 ± 81,841 vs 542,407 ± 51,027 ng/mL; p<0.001), RBP-4 (36.66 ± 4.08 vs 33.67 ± 3.96 ng/mL; p=0.008), and adipsin (408.97 ± 44.17 vs 361.45 ± 48.39 ng/mL; p<0.001) concentrations were higher in pregnancies with macrosomia.

**Table 8:** Maternal and fetal cord blood metabolic and adipokine profiles according to neonatal macrosomia in the full study cohort.

Variable		Non-Macrosomia (<4000 g) (n = 112)	Macrosomia(≥4000 g) (n = 14)	*p* value
Maternal biomarkers				
Glucose (mg/dL)		76.24 ± 13.37	82.55 ± 10.68	0.072
Insulin (µIU/mL)		16.16 ± 16.58	18.65 ± 20.52	0.658
HOMA-IR		3.36 ± 3.89	3.96 ± 4.48	0.396
Total cholesterol (mg/dL)		217.06 ± 57.91	194.09 ± 71.78	0.274
LDL cholesterol (mg/dL)		107.94 ± 41.14	104.09 ± 38.78	0.827
HDL cholesterol (mg/dL)		61.24 ± 15.46	55.45 ± 19.61	0.316
LDL/HDL ratio		1.80 ± 0.64	2.02 ± 0.99	0.516
Triglycerides (mg/dL)		231.89 ± 111.17	220.14 ± 91.30	0.927
Adiponectin (ng/mL)		7,260 ± 120	7,250 ± 160	0.875
Leptin (pg/mL)		41,800 ± 28,760	44,780 ± 28,220	0.593
Asprosin (ng/mL)		10.58 ± 9.69	8.73 ± 5.85	0.831
GLUT-1 (ng/mL)		2.61 ± 1.94	2.41 ± 1.81	0.565
Fetuin-A (ng/mL)		542,407 ± 51,027	631,101 ± 81,841	***<0.001***
RBP-4 (ng/mL)		33.67 ± 3.96	36.66 ± 4.08	***0.008***
Adipsin (ng/mL)		361.45 ± 48.39	408.97 ± 44.17	***<0.001***
Fetal cord blood biomarkers				
Glucose (mg/dL)		60.80 ± 15.69	63.97 ± 19.62	0.636
Insulin (µIU/mL)		4.65 ± 3.95	5.08 ± 5.01	0.887
HOMA-IR		0.83 ± 1.05	0.91 ± 1.14	0.911
Total cholesterol (mg/dL)		70.06 ± 32.78	81.14 ± 53.67	0.831
LDL cholesterol (mg/dL)		33.77 ± 18.76	41.95 ± 35.62	0.923
HDL cholesterol (mg/dL)		27.43 ± 12.71	30.19 ± 13.60	0.831
LDL/HDL ratio		1.72 ± 1.67	1.47 ± 1.06	0.710
Triglycerides (mg/dL)		39.47 ± 57.21	47.36 ± 58.50	0.729
Adiponectin (ng/mL)		27,970 ± 330	27,480 ± 2,910	0.349
Asprosin (ng/mL)		10.26 ± 5.61	7.80 ± 4.67	0.054
Leptin (pg/mL)		16,498 ± 1,997	20,329 ± 3,251	***<0.001***
Fetuin-A (ng/mL)		779,298 ± 58,857	847,611 ± 44,987	***<0.001***
RBP-4 (ng/mL)		12.25 ± 1.39	14.89 ± 2.10	***<0.001***
Adipsin (ng/mL)		179.84 ± 13.64	204.61 ± 9.75	***<0.001***
GLUT-1 (ng/mL)		6.92 ± 2.95	9.32 ± 1.89	*0.001*

Data are presented as mean ± standard deviation (SD). A two-sided *p* < 0.05 was considered statistically significant. Significant *p* values are indicated in bold italics. HDL, high-density lipoprotein; LDL, low-density lipoprotein; HOMA-IR, homeostatic model assessment of insulin resistance; RBP-4, retinol-binding protein-4; GLUT-1, glucose transporter-1; SD, standard deviation.

Fetal concentrations of leptin (20,329 ± 3,251 vs 16,498 ± 1,997 pg/mL), fetuin-A (847,611 ± 44,987 vs 779,298 ± 58,857 ng/mL), RBP-4 (14.89 ± 2.10 vs 12.25 ± 1.39 ng/mL), adipsin (204.61 ± 9.75 vs 179.84 ± 13.64 ng/mL), and GLUT-1 (9.32 ± 1.89 vs 6.92 ± 2.95 ng/mL) were significantly higher in macrosomic than in non-macrosomic neonates (all p ≤ 0.001).

### Diagnostic performance of maternal and fetal cord blood biomarkers for neonatal macrosomia

**Figure 6:**
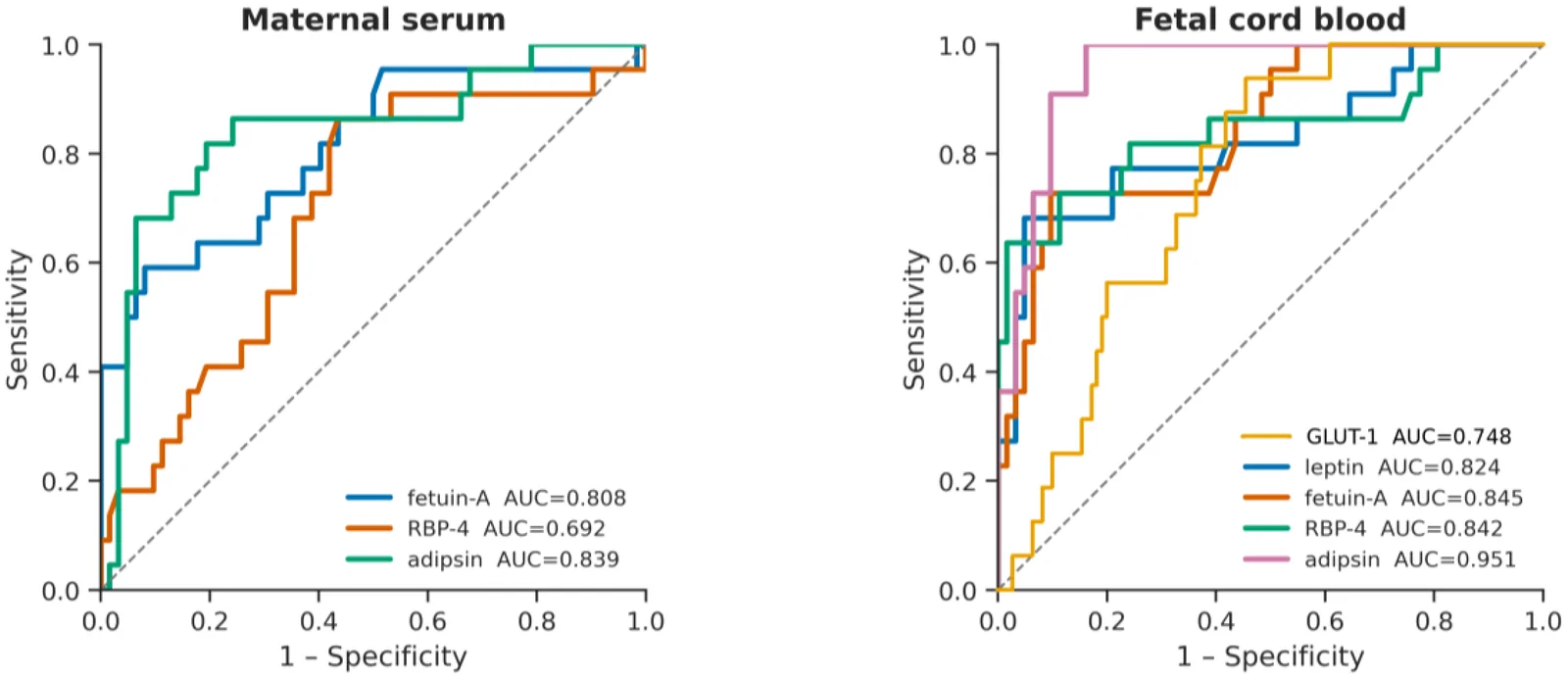
Receiver operating characteristic curves for selected maternal and fetal biomarkers and neonatal macrosomia. Fetal adipsin showed the highest AUC among the evaluated fetal biomarkers.

ROC findings for macrosomia are summarized in **Table 9** and **Figure 6**. Among maternal biomarkers, adipsin showed the highest discriminatory performance (AUC = 0.839), followed by fetuin-A (AUC = 0.808) and RBP-4 (AUC = 0.692). Among fetal biomarkers, adipsin showed the highest discriminatory performance (AUC = 0.951), followed by fetuin-A (AUC = 0.845), RBP-4 (AUC = 0.842), leptin (AUC = 0.824), and GLUT-1 (AUC = 0.748).

**Table 9:** Discriminatory performance of maternal and fetal cord blood biomarkers for neonatal macrosomia.

Biomarker	Source	Cut-off	Sensitivity (%)	Specificity (%)	AUC (95% CI)	*p* value
Adipsin	Maternal serum	>384.49	81.8	80.6	0.839 (0.729-0.937)	***<0.001***
Fetuin-A	Maternal serum	>617,100	59.1	91.9	0.808 (0.686-0.908)	***<0.001***
RBP-4	Maternal serum	>34.22	86.4	56.5	0.692 (0.564-0.816)	***0.006***
Leptin	Fetal cord blood	>20,054	68.2	95.2	0.824 (0.699-0.926)	***<0.001***
Adipsin	Fetal cord blood	>194.17	100.0	83.9	0.951 (0.907-0.985)	***<0.001***
Fetuin-A	Fetal cord blood	>826,362	72.7	90.3	0.845 (0.749-0.929)	***<0.001***
RBP-4	Fetal cord blood	>15.40	63.6	98.4	0.842 (0.718-0.945)	***<0.001***
GLUT-1	Fetal cord blood	>7.00	93.8	54.5	0.748 (0.645-0.842)	***<0.001***

Data are derived from receiver operating characteristic (ROC) curve analyses evaluating the discriminatory performance of maternal and fetal biomarkers for neonatal macrosomia. For clarity, only biomarkers demonstrating statistically significant or clinically relevant discriminatory performance are presented. A two-sided *p* < 0.05 was considered statistically significant. Significant *p* values are indicated in bold italics. AUC, area under the curve; CI, confidence interval; GLUT-1, glucose transporter-1; RBP-4, retinol-binding protein-4.

### Multivariable logistic regression model for neonatal macrosomia

As shown in **Table 10** and **Figure 5**, fetal adipsin remained statistically associated with macrosomia in the exploratory multivariable model (adjusted OR = 5.425 per 10-ng/mL increase, 95% CI 2.138-13.769; p<0.001). In contrast, maternal fetuin-A and fetal GLUT-1 were no longer statistically significant after simultaneous adjustment (adjusted OR = 1.062 per 10,000-ng/mL increase, 95% CI 0.924-1.221; p=0.398 and adjusted OR = 1.157 per 1-ng/mL increase, 95% CI 0.868-1.543; p=0.320, respectively). Given the limited number of macrosomia events and the wide confidence interval for fetal adipsin, these adjusted estimates should be interpreted cautiously.

**Table 10:** Exploratory multivariable logistic regression analysis for neonatal macrosomia.

Predictor	Unadjusted OR (95% CI)	*p* value	Adjusted OR (95% CI)	*p* value
Pre-pregnancy BMI	–	–	0.926 (0.760-1.129)	0.447
Maternal fetuin-A (per 10,000 ng/mL)	1.251 (1.118-1.398)	***<0.001***	1.062 (0.924-1.221)	0.398
Fetal adipsin (per 10 ng/mL)	28.016 (4.179-187.780)	***<0.001***	5.425 (2.138-13.769)	***<0.001***
Fetal GLUT-1 (per 1 ng/mL)	1.353 (1.102-1.661)	***0.004***	1.157 (0.868-1.543)	0.320

Values are odds ratios (95% confidence intervals), followed by p values. The multivariable macrosomia model included pre-pregnancy BMI, maternal fetuin-A, fetal adipsin, and fetal GLUT-1. Biomarker odds ratios are expressed per increment stated in the table. A two-sided *p* < 0.05 was considered statistically significant. BMI, body mass index; CI, confidence interval; OR, odds ratio. Bold values indicate statistical significance at p < 0.05.

## Discussion

Maternal obesity is increasingly recognized not merely as excessive maternal adiposity, but as a chronic low-grade inflammatory and metabolically dysregulated state characterized by altered adipokine profiles, insulin resistance, lipotoxicity, and placental dysfunction. Previous studies have demonstrated that obese pregnancies are associated with enhanced transplacental nutrient transport, increased placental inflammatory activity, and altered fetal metabolic homeostasis. Catalano et al. previously proposed that fetuses of obese mothers may develop insulin resistance *in utero*, thereby contributing to the concept of early fetal metabolic programming (18). Similarly, Brombach et al. highlighted placental metabolic adaptations in maternal obesity involving glucose transport, lipid handling, inflammatory signaling, and endothelial dysfunction (19). Our findings are consistent with these previous observations by demonstrating simultaneous associations between maternal obesity severity and multiple maternal and fetal cord blood adipokine alterations.

The present analysis demonstrated significant increases in maternal fetuin-A, RBP-4, and adipsin, as well as fetal leptin, fetuin-A, RBP-4, adipsin, and GLUT-1, with increasing maternal obesity severity. The frequencies of macrosomia and NICU requirement also increased across obesity severity groups. In exploratory multivariable analyses, fetal adipsin remained associated with macrosomia, and fetal RBP-4 remained associated with NICU requirement. Because of the limited number of outcome events and the correlations among the included predictors, these findings should not be interpreted as evidence of independently validated predictors. Nevertheless, the observed maternal and fetal biomarker patterns suggest that increasing maternal obesity severity is accompanied by broader metabolic alterations across the maternal-fetal unit, which may contribute to the higher burden of adverse neonatal outcomes observed in these pregnancies.

The significantly higher NICU admission rate observed in class II-III obesity suggests that severe maternal obesity is associated not only with altered fetal growth but also with early neonatal clinical outcomes. These observations are consistent with previous epidemiological studies demonstrating increased neonatal respiratory morbidity, transient tachypnea, hypoglycemia, and metabolic instability in neonates born to mothers with obesity (20). Dammer et al. similarly reported increased perinatal morbidity and NICU transfer rates in pregnancies complicated by elevated preconceptional BMI (21). However, our findings extend these previous observations by demonstrating that adverse neonatal outcomes in pregnancies complicated by maternal obesity occur alongside distinct alterations in maternal and fetal cord blood adipokine profiles. This finding provides a basis for examining whether specific components of these altered biomarker profiles may help explain the metabolic pathways linking maternal obesity to neonatal outcomes.

An important aspect of the present study is the simultaneous characterization of maternal and fetal cord blood biomarker profiles across increasing degrees of maternal obesity. Previous studies have primarily focused on classical adipokines, particularly leptin and adiponectin, and have demonstrated alterations in their maternal and cord blood concentrations in relation to maternal obesity and neonatal anthropometric measures (22). By incorporating fetuin-A, RBP-4, adipsin, asprosin, and GLUT-1 alongside these established adipokines, our study provides a broader assessment of the metabolic changes occurring across the maternal–fetal unit and allows these less extensively studied biomarkers to be considered within the same biological context. The observed concentrations and obesity-related patterns were generally consistent with available maternal–fetal studies, although the extent of comparability varied among biomarkers because of differences in assay platforms, sample characteristics, and reporting units (13, 16, 22, 23). Representative values from previous studies are therefore provided in **Supplementary Table 2** to place the present findings within the range of published maternal and cord blood concentrations. Taken together, the simultaneous evaluation of these biomarkers suggests that the metabolic effects accompanying maternal obesity extend beyond alterations in classical adipokines and involve a broader network of maternal and fetal metabolic signals that may be relevant to neonatal phenotype.

Among maternal biomarkers, fetuin-A demonstrated one of the most evident obesity-associated increases and was associated with both macrosomia and NICU requirement in unadjusted analyses; however, these associations were attenuated after simultaneous adjustment in the reported multivariable models. Fetuin-A is a hepatokine associated with insulin resistance, systemic inflammation, ectopic lipid accumulation, and impaired insulin receptor signaling. Experimental studies have shown that fetuin-A suppresses insulin receptor tyrosine kinase activity and amplifies inflammatory pathways through toll-like receptor signaling (24, 25). Its progressive elevation with increasing obesity severity may therefore reflect escalating hepatometabolic stress during pregnancy; however, the present observational data do not establish a causal effect on neonatal outcomes.

RBP-4 showed a distinct maternal–fetal pattern across increasing obesity severity, with concentrations rising in both maternal serum and fetal cord blood. This finding is biologically relevant because RBP-4 is closely linked to insulin resistance and altered glucose metabolism, and previous pregnancy studies have associated elevated RBP-4 concentrations with gestational diabetes mellitus, excessive fetal growth, and disturbances in fetal metabolic homeostasis (26–28). The progressive increase in fetal cord blood RBP-4 observed in our cohort is also consistent with reports by Yang et al. and Chan et al., who demonstrated higher cord blood RBP-4 concentrations in relation to fetal overgrowth (29, 30). Importantly, fetal RBP-4 remained statistically associated with NICU requirement after adjustment, distinguishing it from several other evaluated biomarkers whose associations were attenuated in the multivariable model. This finding suggests that fetal RBP-4 may capture a component of neonatal metabolic vulnerability that is not fully reflected by maternal BMI or the other biomarkers included in the model and supports further investigation of cord blood RBP-4 as a marker of early neonatal risk in pregnancies complicated by maternal obesity.

Adipsin displayed a clear obesity-related gradient in both maternal serum and fetal cord blood, suggesting that this adipokine may be particularly responsive to the metabolic changes accompanying increasing maternal adiposity. Adipsin, also known as complement factor D, is an adipose tissue-derived component of the alternative complement pathway with important links to metabolic homeostasis. Particularly relevant to pregnancy, Sivakumar et al. demonstrated increased fetal adipsin concentrations in pregnancies complicated by maternal obesity, with fetal levels showing positive correlations with maternal BMI as well as maternal and fetal insulin resistance; they further identified placental Hofbauer cells as a source of adipsin and demonstrated greater placental adipsin secretion in obese pregnancies (31). These observations provide a biologically relevant context for the parallel increases in maternal and fetal adipsin observed in our cohort and suggest that the fetal adipsin profile may reflect metabolic changes extending beyond maternal adipose tissue to the fetoplacental compartment. Of particular interest, fetal adipsin showed the strongest discriminatory performance for macrosomia among the evaluated biomarkers and remained significantly associated with macrosomia after multivariable adjustment. This finding links the progressive rise in fetal adipsin with excessive fetal growth and raises the possibility that cord blood adipsin captures aspects of fetal metabolic adaptation to maternal obesity that are particularly relevant to fetal growth. Together, these observations support fetal adipsin as a promising biomarker for further investigation in obesity-associated fetal overgrowth.

Asprosin showed a different pattern from fetuin-A, RBP-4, and adipsin, with no significant variation across obesity severity groups in either maternal or fetal circulation. As a fasting-induced glucogenic adipokine, asprosin promotes hepatic glucose release through activation of the G protein-cAMP-PKA pathway and has been implicated in insulin resistance and glucose homeostasis (32). Its role during pregnancy, however, appears to be more closely related to the prevailing metabolic and glycemic environment than to maternal adiposity alone. Previous studies have reported increased maternal and fetal asprosin concentrations in pregnancies complicated by gestational diabetes and other metabolically adverse pregnancy phenotypes, whereas associations with maternal BMI have been less consistent. The absence of an obesity-dependent gradient in our cohort, despite clear increases in several other maternal and fetal biomarkers, therefore, suggests that circulating asprosin may respond to metabolic disturbances that are not captured simply by increasing obesity severity. This distinct pattern also underscores the heterogeneity of adipokine responses to maternal obesity and suggests that different components of adipose-derived signaling may reflect complementary rather than parallel aspects of maternal-fetal metabolic adaptation.

Fetal GLUT-1 findings warrant particular consideration in the context of maternal-fetal glucose metabolism. Although cord-blood GLUT-1 concentrations varied with maternal obesity severity and neonatal outcomes, these associations were attenuated after multivariable adjustment, suggesting that circulating GLUT-1 may largely reflect the broader metabolic milieu accompanying maternal obesity rather than provide distinct information regarding neonatal outcomes. This interpretation is biologically plausible given the central role of GLUT-1 in placental glucose handling. GLUT-1 is abundantly expressed in the syncytiotrophoblast and represents a major component of the facilitated glucose transport system across the maternal-fetal interface (14, 33). However, the biological significance of circulating GLUT-1 in fetal cord blood remains less well established, and its concentration should not be considered a surrogate for placental GLUT-1 expression, membrane localization, or transport activity. The obesity-related variation in cord-blood GLUT-1 observed in our cohort may therefore reflect alterations in maternal-placental-fetal glucose homeostasis without necessarily representing an independent pathway linking maternal obesity to neonatal outcome. Studies integrating maternal and cord-blood measurements with placental GLUT-1 expression and functional assessment of glucose transport are needed to clarify the biological basis and potential clinical relevance of these findings.

A different pattern emerged when the remaining metabolic parameters were considered. Among the classical adipokines, fetal leptin increased progressively with obesity severity, whereas maternal adiponectin showed a modest decrease and maternal leptin and fetal adiponectin did not differ significantly across groups. Similarly, several conventional metabolic parameters, including glucose, insulin, HOMA-IR, LDL cholesterol, and triglycerides, did not show the progressive obesity-related gradients observed for fetuin-A, RBP-4, adipsin, and fetal GLUT-1. These contrasting patterns suggest that the biomarker changes identified in our cohort cannot be explained simply by a generalized increase in circulating metabolic markers with increasing BMI. Instead, maternal obesity appears to be accompanied by selective alterations in specific metabolic pathways involving adipose tissue, hepatic metabolism, inflammatory signaling, and fetal metabolic adaptation (34), with distinct responses in the maternal and fetal compartments. The progressive changes in fetal leptin, RBP-4, adipsin, and GLUT-1 are particularly notable in this regard, as they indicate that increasing maternal adiposity is accompanied by a distinct fetal metabolic response rather than merely mirroring changes in the maternal circulation.

Whether this distinct fetal metabolic response has implications beyond the immediate neonatal period is an important question raised by our findings. Developmental origins and metabolic programming models propose that exposure to an adverse intrauterine metabolic environment may have lasting effects on offspring metabolic regulation and subsequent cardiometabolic health (35–37). In this context, the coordinated alterations in fetal leptin, RBP-4, adipsin, and GLUT-1 observed with increasing maternal obesity severity may provide a biochemical link between the intrauterine metabolic environment and the neonatal phenotype identified in our cohort. Although the present study was not designed to establish fetal metabolic programming or its long-term consequences, these findings provide a rationale for investigating whether such fetal biomarker patterns persist beyond birth or identify children with greater subsequent metabolic vulnerability. Longitudinal studies integrating maternal and fetal biomarker profiles with placental, epigenetic, and childhood metabolic assessments will be important to determine whether these early alterations are related to later obesity, insulin resistance, type 2 diabetes mellitus, or cardiovascular risk.

Several features strengthen the present study. The simultaneous evaluation of maternal and fetal biomarker profiles across increasing degrees of obesity allowed characterization of metabolic alterations in both compartments within the same pregnancies, while assessment of macrosomia and NICU requirement linked these profiles to clinically relevant neonatal outcomes. The inclusion of less extensively studied biomarkers, particularly adipsin, fetuin-A, RBP-4, and circulating fetal GLUT-1, alongside classical adipokines provided a broader characterization of obesity-associated maternal-fetal metabolic alterations. Finally, the combined use of ROC analyses and multivariable regression enabled complementary evaluation of biomarker discrimination and adjusted associations with neonatal outcomes.

Nevertheless, several limitations should be acknowledged. First, this single-center study had a moderate sample size, which may limit the generalizability of the findings. Second, the precision of the multivariable estimates for macrosomia was limited by the number of outcome events, as reflected by the wide confidence intervals; these findings should therefore be considered exploratory and warrant confirmation in larger cohorts. In addition, the inverse association between pre-pregnancy BMI and NICU requirement observed after multivariable adjustment should not be interpreted as a protective effect of higher maternal BMI. Given the correlations between BMI and several biomarkers included in the model, this coefficient reversal may reflect shared variance or a suppression effect arising from their simultaneous inclusion. Third, placental tissue analyses, transporter-expression studies, and inflammatory cytokine profiling were not performed; therefore, the underlying placental mechanisms could not be directly investigated. Fourth, only total adiponectin was measured, precluding evaluation of potential differences among adiponectin isoforms. Fifth, NICU admission represented a clinically heterogeneous outcome, and the absence of systematically recorded admission indications and length of stay precluded assessment of biomarker associations with specific neonatal morbidities or illness severity.

In conclusion, increasing maternal obesity severity was accompanied by distinct and compartment-specific alterations in maternal and fetal metabolic profiles, together with progressively higher frequencies of macrosomia and NICU requirement. The coordinated increases in fetal leptin, fetuin-A, RBP-4, adipsin, and GLUT-1 indicate that the metabolic consequences of maternal obesity extend into the fetal circulation, while the differing patterns observed across maternal and fetal compartments suggest that the fetal response is not simply a reflection of maternal metabolic status. Among these biomarkers, fetal adipsin showed particularly strong discrimination for macrosomia and remained associated with excessive fetal growth after multivariable adjustment, whereas fetal RBP-4 remained associated with NICU requirement. These findings identify adipsin and RBP-4 as particularly informative components of the fetal metabolic phenotype accompanying obesity-related neonatal outcomes and provide a rationale for their further evaluation in larger, independent cohorts. More broadly, the maternal-fetal biomarker patterns observed in this study support the concept that increasing maternal adiposity is accompanied by selective alterations in fetal metabolic signaling that may contribute to the biological link between maternal obesity and early neonatal phenotype.

## Data Availability

The raw data supporting the conclusions of this article will be made available by the authors, without undue reservation.

## Supplementary material

Supplementary Table 1 ELISA kit characteristics, assay performance parameters, sample dilution factors, and pre-analytical conditions. This table summarizes the commercially available human ELISA kits used for maternal and fetal serum analyses. Samples were diluted at the analyte- and compartment-specific ratios listed above prior to measurement. For samples yielding concentrations above the upper limit of the standard curve at the prespecified dilution, the assay was repeated using a higher dilution factor, and the corresponding dilution factor was applied when calculating the final concentration in the original sample. Samples were thawed once immediately before analysis, and repeated freeze-thaw cycles were avoided. Because inter-assay variability, dilution protocol, and methodological heterogeneity may limit direct comparison with external studies, absolute concentrations and ROC-derived cut-off points should be interpreted as exploratory and require confirmation in independent cohorts using additional analytical platforms. The dilution-adjusted final reportable range was calculated by multiplying both limits of the assay standard-curve range by the applicable maternal or fetal dilution factor. For samples requiring a higher dilution factor, the corresponding dilution-adjusted reportable range was applied. Abbreviations: CV, coefficient of variation; CFD, complement factor D; GLUT-1, glucose transporter-1; RBP-4, retinol-binding protein-4; ADP/ACRP30, adiponectin; FETUA, fetuin-A; INS, insulin; LEP, leptin.

Supplementary Table 2 Representative published concentration values and interpretive context for evaluated biomarkers. These values are representative examples and are not universal reference intervals. Assay platform, antibody specificity, calibration procedure, sample matrix, gestational timing, storage conditions, and dilution protocol can substantially influence absolute concentrations.
