# Supplementary material for: Maternal-fetal adipokine remodeling in obesity-complicated pregnancies: associations with neonatal macrosomia and NICU requirement
Source: Front Endocrinol (Lausanne). 2026 Sep 9;17:1904956. doi: 10.3389/fendo.2026.1904956 (PMC13597342; doi:10.3389/fendo.2026.1904956)
Supplement: Supplementary file 1 [file Table1.pdf]

Supplementary Material

Supplementary Tables S1 and S2 summarize the ELISA kit characteristics, assay performance parameters, and pre-analytical conditions used for all biomarker measurements, and provide representative published concentration ranges for contextual interpretation.

Supplementary Table S1. ELISA kit characteristics, assay performance parameters, sample dilution factors, and pre-analytical conditions

Biomarker	Full kit name	Catalog no.	Assay principle	Detection range	Dilution factor (maternal / fetal)	Intra-assay CV	Inter-assay CV	Dilution-adjusted reportable range (maternal / fetal)
**Adipsin / CFD**	Human CFD / Complement Factor D ELISA Kit	ABT2732Hu	Competitive ELISA	0.56-100 ng/mL	1:20 / 1:20	<10%	<10%	11.2-2,000 / 11.2-2,000 ng/mL
**Total adiponectin / ADP-Acrp30**	Human ADP/Acrp30 Adiponectin ELISA Kit	ABT2837Hu	Sandwich ELISA	31.25-2000 ng/mL	1:5 / 1:20	≤10%	≤10%	156.25-10,000 / 625-40,000 ng/mL
**Fetuin-A / FETUA**	Human FETUA / Fetuin-A ELISA Kit	ABT1211Hu	Sandwich ELISA	9.38-600 ng/mL	1:2000 / 1:2000	≤10%	≤10%	18,760-1,200,000 / 18,760-1,200,000 ng/mL
**Asprosin / ASP**	Human ASP / Asprosin ELISA Kit	ABT3703Hu	Sandwich ELISA	0.16-10 ng/mL	1:3 / 1:3	≤10%	≤10%	0.48-30 / 0.48-30 ng/mL
**GLUT-1**	Human GLUT-1 / Glucose Transporter-1 ELISA Kit	ABT2059Hu	Sandwich ELISA	0.16-10 ng/mL	1:2 / 1:2	<10%	<10%	0.32-20 / 0.32-20 ng/mL
**Insulin / INS**	Human INS / Insulin ELISA Kit	ABT3153Hu	Sandwich ELISA	1.56-100 µIU/mL	1:1 / 1:1	<10%	<10%	1.56-100 / 1.56-100 µIU/mL
**Leptin / LEP**	Human LEP / Leptin ELISA Kit	ABT2739Hu	Sandwich ELISA	15.63-1000 pg/mL	1:100 / 1:100	<10%	<10%	1,563-100,000 / 1,563-100,000 pg/mL
**RBP-4**	Human RBP4 / Retinol-Binding Protein 4 ELISA Kit	ABT3065Hu	Sandwich ELISA	1.57-100 ng/mL	1:5 / 1:1	≤10%	≤10%	7.85-500 / 1.57-100 ng/mL

This table summarizes the commercially available human ELISA kits used for maternal and fetal serum analyses. Samples were diluted at the analyte- and compartment-specific ratios listed above prior to measurement. For samples yielding concentrations above the upper limit of the standard curve at the prespecified dilution, the assay was repeated using a higher dilution factor, and the corresponding dilution factor was applied when calculating the final concentration in the original sample. Samples were thawed once immediately before analysis, and repeated freeze-thaw cycles were avoided. Because inter-assay variability, dilution protocol, and methodological heterogeneity may limit direct comparison with external studies, absolute concentrations and ROC-derived cut-off points should be interpreted as exploratory and require confirmation in independent cohorts using additional analytical platforms. The dilution-adjusted final reportable range was calculated by multiplying both limits of the assay standard-curve range by the applicable maternal or fetal dilution factor. For samples requiring a higher dilution factor, the corresponding dilution-adjusted reportable range was applied. Abbreviations: CV, coefficient of variation; CFD, complement factor D; GLUT-1, glucose transporter-1; RBP-4, retinol-binding protein-4; ADP/ACRP30, adiponectin; FETUA, fetuin-A; INS, insulin; LEP, leptin.
