# Supplementary material for: Maternal-fetal adipokine remodeling in obesity-complicated pregnancies: associations with neonatal macrosomia and NICU requirement
Source: Front Endocrinol (Lausanne). 2026 Sep 9;17:1904956. doi: 10.3389/fendo.2026.1904956 (PMC13597342; doi:10.3389/fendo.2026.1904956)
Supplement: Supplementary file 2 [file Table2.pdf]

Supplementary Table S2. Representative published concentration values and interpretive context for evaluated biomarkers

Biomarker	Representative published values	Context/sample	Unit	Source
Leptin	Maternal 37.17 ± 28.07; cord 14.78 ± 15.97. Another paired study: maternal 27.3 ± 36.5; fetal 5.6 ± 4.8.	Term pregnancy; maternal/cord or fetal blood	ng/mL	Stefaniak 2019; Solis-Paredes 2016
Adiponectin	Maternal 7.3 ± 4.8; fetal 28.0 ± 15.1.	Term paired maternal-fetal adipokine profiles	µg/mL	Solis-Paredes 2016; Downs 2024
RBP-4	Maternal delivery ≈30.5 ± 10.4; cord ≈10.9 ± 3.8; upper fetal-growth quartile cord ≈14.3 ± 3.7.	GDM/control pregnancy and fetal-growth studies	ng/mL	Chan 2007; Chan 2011; Yang 2020
Adipsin / ASP	Cord adipsin 1663.78 ± 52.76 pg/mL; cord ASP 354.48 ± 17.17 ng/mL; maternal ASP 608.70 ± 67.15 vs 420.30 ± 39.98.	Obese vs normal-BMI pregnancy; cord and maternal plasma	pg/mL; ng/mL	Sivakumar 2013
Fetuin-A	Cord fetuin-A 783.6 ± 320.0 in GDM and 754.8 ± 281.9 in euglycemic pregnancies.	Cord blood; GDM vs euglycemic pregnancies	µg/mL	Wang 2020
Asprosin	Maternal range 2.93-21.64, mean 8.11 ± 3.80; fetal range 1.91-20.13, mean 9.60 ± 3.87.	Maternal and fetal umbilical plasma	ng/mL	Hoffmann 2022
GLUT-1	Standardized circulating serum ranges are scarce; most pregnancy studies report placental GLUT-1 expression.	Placental tissue/expression; limited circulating serum data	Expression-based	Borges 2019; Stanirowski 2018

These values are representative examples and are not universal reference intervals. Assay platform, antibody specificity, calibration procedure, sample matrix, gestational timing, storage conditions, and dilution protocol can substantially influence absolute concentrations.

References for Supplementary Table S2

1. Stefaniak M, Dmoch-Gajzlerska E, Mazurkiewicz B, Gajzlerska-Majewska W. Maternal serum and cord blood leptin concentrations at delivery. PLoS One. 2019;14(11):e0224863.

2. Solis-Paredes M, Espino Y Sosa S, Estrada-Gutierrez G, Nava-Salazar S, Ortega-Castillo V, et al. Maternal and fetal lipid and adipokine profiles and their association with obesity. International Journal of Endocrinology. 2016;2016:7015626.

3. Downs T, da Silva Costa F, de Freitas Paganoti C, Holland OJ, Hryciw DH. Adiponectin and leptin during pregnancy: a systematic review of their association with pregnancy disorders, fetal growth and placental function. Endocrines. 2024;5(3):382–394.

4. Chan TF, Chen HS, Chen YC, Lee CH, Chou FH, Chen IJ, et al. Increased serum retinol-binding protein 4 concentrations in women with gestational diabetes mellitus. Reproductive Sciences. 2007;14(2):169-174.

5. Chan TF, Tsai YC, Wu CH, Lee CH, Wang SH, Su JH. The positive correlation between cord serum retinol-binding protein 4 concentrations and fetal growth. Gynecologic and Obstetric Investigation. 2011;72(2):98-102.

6. Yang MN, Zhang GH, Du K, Wang WJ, Dong Y, He H, et al; Shanghai Birth Cohort. Retinol-binding protein 4, fetal overgrowth and fetal growth factors. Pediatric Research. 2020;87(5):946-951.

7. Sivakumar K, Bari MF, Adaikalakoteswari A, Guller S, Weickert MO, Randeva HS, et al. Elevated fetal adipsin/acylation-stimulating protein (ASP) in obese pregnancy: novel placental secretion via Hofbauer cells. J Clin Endocrinol Metab. 2013;98(10):4113-22.

8. Wang WJ, Zhang L, Zheng T, Zhang GH, Du K, Chiu HC, et al. Fetuin-A and fetal growth in gestational diabetes mellitus. BMJ Open Diabetes Res Care. 2020;8(1):e000864.

9. Hoffmann T, Morcos YAT, Janoschek R, Turnwald EM, Gerken A, Müller A, Sengle G, Dötsch J, Appel S, Hucklenbruch-Rother E. Correlation of metabolic characteristics with maternal, fetal and placental asprosin in human pregnancy. Endocr Connect. 2022;11(3):e220069.

10. Borges MH, Pullockaran J, Catalano PM, Baumann MU, Zamudio S, Illsley NP. Human placental GLUT1 glucose transporter expression and the fetal insulin-like growth factor axis in pregnancies complicated by diabetes. Biochim Biophys Acta Mol Basis Dis. 2019;1865(9):2411-9.

11. Stanirowski PJ, Szukiewicz D, Pazura-Turowska M, Sawicki W, Cendrowski K. Placental expression of glucose transporter proteins in pregnancies complicated by gestational and pregestational diabetes mellitus. Canadian Journal of Diabetes. 2018;42(2):209–217.
